# Non-vascularized Fibular Autograft and Locking Plate: A Solution to the Resistant Nonunion of a Tibial Metaphyseal Fracture

**DOI:** 10.7759/cureus.58831

**Published:** 2024-04-23

**Authors:** Andrei Morcovescu, Horea Pop, Maria M Mihai, Andrei S Constantinescu, Matei Gheorghiu Branaru, Radu Paraschiv, Razvan M Vlad, Florin Bica

**Affiliations:** 1 Orthopedics and Traumatology, Bagdasar-Arseni Emergency Clinical Hospital, Bucharest, ROU; 2 Plastic and Reconstructive Surgery, Bagdasar-Arseni Emergency Clinical Hospital, Bucharest, ROU; 3 Orthopedics and Traumatology, Carol Davila University of Medicine and Pharmacy, Bucharest, ROU

**Keywords:** proximal tibia fracture, fractures of the tibia, iliac crest bone graft, non-vascularized fibula graft, intramedullary nailing of the tibia, nonunion of tibia, aseptic nonunion

## Abstract

The union of tibial fractures often raises concerns. In one such case, a 62-year-old female patient presented in our Emergency Room (ER) with a comminuted tibial metaphyseal fracture resulting from a traffic accident. The patient underwent three surgical procedures in the following two years. The first intervention was open reduction internal fixation (ORIF) with a plate and screws. The second intervention, which took place three months after the first surgery, addressed the union delay through implant removal and closed reduction and internal fixation (CRIF) with an antegrade intramedullary tibial nail. The third intervention addressed the implant failure and oligotrophic nonunion through the removal of the broken tibial nail and ORIF using a proximal tibia locking plate and screws, augmented with fibular shaft and reamed iliac crest autografts. We conducted frequent follow-ups with the patient and performed multiple X-rays to confirm and monitor the fracture union. At the last follow-up, two years after the last surgical intervention, imagistic investigations showed that the patient presented with fracture union, she could support her full body weight on the operated leg, and was able to walk and carry out normal daily activities. As such, we concluded that the surgical method chosen (ORIF with proximal tibia locking plate and screws, augmented with a fibula shaft strut and reamed iliac crest autograft) was a viable option to treat an aseptic oligotrophic nonunion in a high-energy comminuted tibia fracture.

## Introduction

The tibia is one of the most often fractured long bones of the human body; it also has a high rate of malunion and nonunion: with the rate of nonunion sitting between 1 and 8% for surgically treated closed tibia fractures [1]. Tibia fractures may result either from an indirect trauma in a low-energy mechanism (such as torsion, yielding spiral fractures) or direct trauma in a high-energy event (which leads to oblique or wedge fractures with marked comminution) [2]. The effect on the soft tissue should also be taken into account; there is likely to be negligible soft-tissue damage resulting from low-energy events compared to marked soft-tissue injury in high-energy trauma [2] such as vehicular trauma.

Nonunion rates in tibia fractures are higher in the proximal third of the bone than in the midshaft [2]. This may be due to poor soft tissue coverage or the nature of the traumatic forces affecting this area of the tibia [2]. The treatment of tibial fracture nonunions is challenging; this challenge can be exacerbated by patient age, osteoporotic bones, the presence of diabetes, comminution, and obesity [3]. Nonunion means that there is no sign of bone union at six to eight months in simple fractures or at eight to nine months in fractures with comminution [4]. Other texts describe nonunion as a lack of bone healing that is visible on X-rays three months after the fracture and a lack of consolidation nine months after the fracture [5]. The tibia can show either hypertrophic nonunion, in which a callus forms but does not unify, often caused by inefficient internal fixation or the use of conservative treatment; atrophic nonunion, in which a callus does not form, often caused by bone devascularization brought about by the trauma or through damage to periosteum caused during the plating process [4]; or oligotrophic nonunion, which is indicated by poor or no callus formation but with still-viable bone fragments [5]. Pseudarthrosis is considered the end stage of nonunion, indicated by the presence of an articular capsule [5].

The patient should be urgently investigated with anteroposterior (AP) and lateral X-rays of the knee, entire leg, and ankle. A CT scan should be ordered if articular involvement is suspected.

There are various means of classifying tibia shaft fractures, such as the AO Foundation/Orthopaedic Trauma Association (AO/OTA) fracture classification, the Tscherne classification for closed fractures, and the Gustilo classification for open fractures [1].

Tibia fractures can be tackled in several ways; options range from non-operative treatment in patients who have a high anesthetic risk, refuse surgical treatment, or have excellent alignment in the splint or cast (although this is very much a non-preferred treatment option) [1]; or surgical treatment with an intramedullary nail, plating, or an external fixator [1]. Each of these options has its drawbacks; intramedullary nailing risks proximal or distal fracture malalignment and postoperative knee pain, while plating risks bone devascularization and external fixation risks infection on the pin tract, nonunion, and a loss of alignment [1].

The purpose of this case report is to show that the use of a fibular shaft strut autograft and reamed iliac crest autograft, when performed in combination with a locking plate and screws, was a viable solution to an oligotrophic aseptic nonunion in a comminuted tibial metaphyseal fracture.

## Case presentation

Our study follows the post-operative evolution of a 62-year-old female patient with a high-energy comminuted tibial metaphyseal fracture. This fracture led to oligotrophic nonunion and implant failure but was eventually successfully treated with fibula and reamed iliac crest autografts and a proximal tibia locking plate. Informed consent was obtained from the patient regarding this study, and the institutional review board granted its approval.

**Figure 1:**
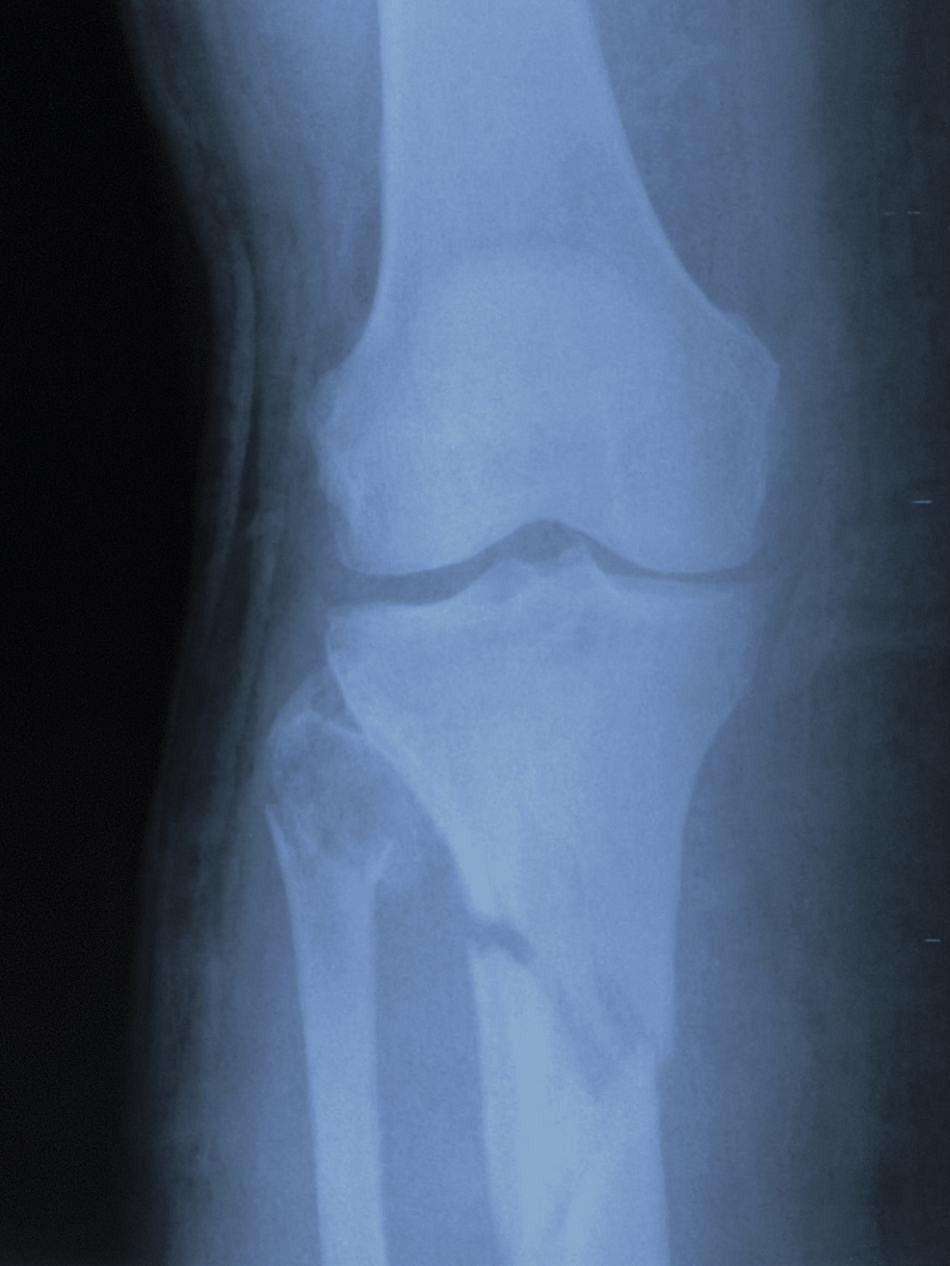
Anteroposterior X-ray of the right knee and proximal leg showing comminuted tibial metaphyseal fracture and proximal fibula fracture

**Figure 2:**
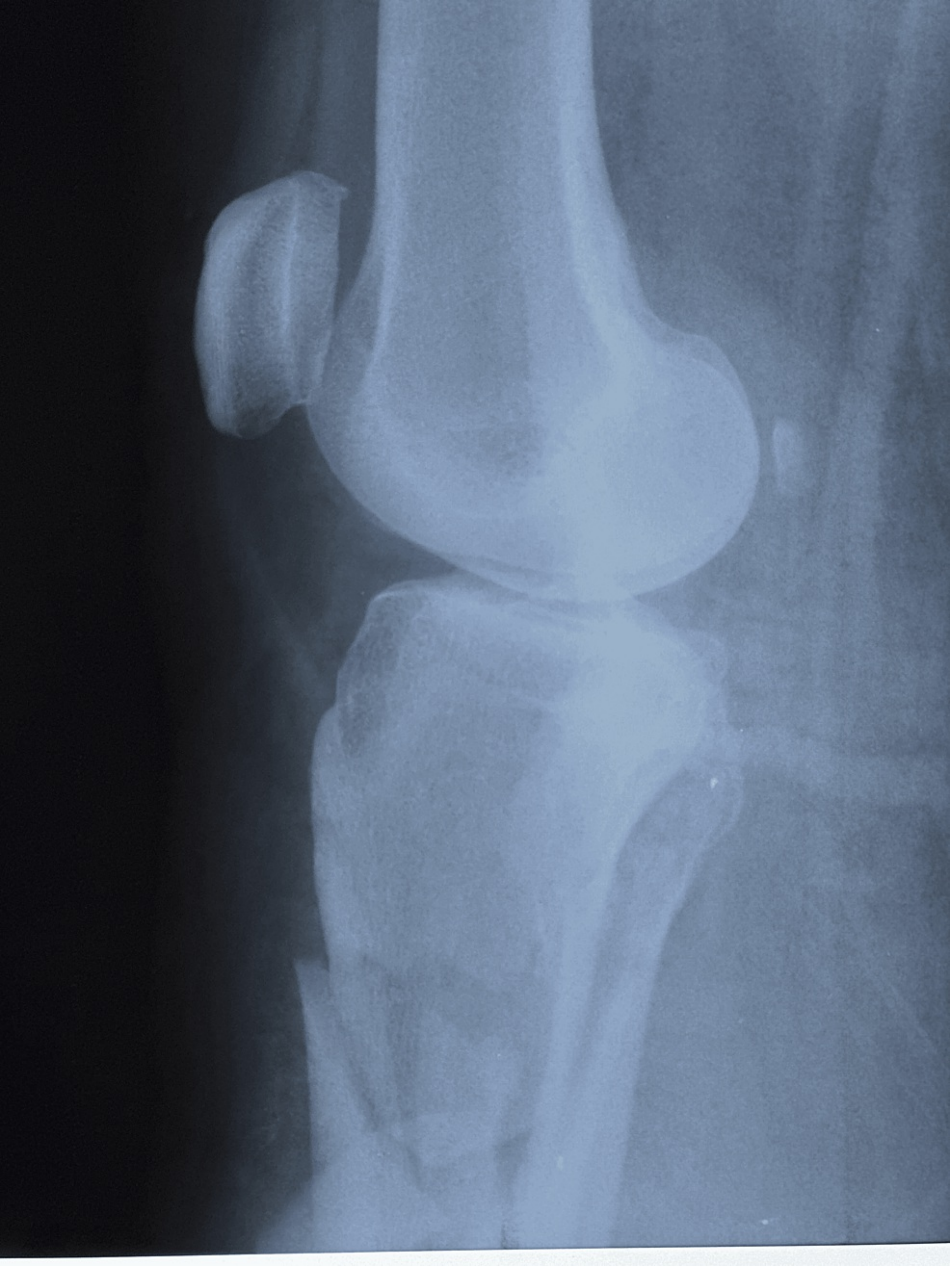
Lateral X-ray of the right knee and proximal right leg showing tibial metaphyseal comminuted fracture and fracture of the proximal fibula

The patient was brought to the Emergency Room after suffering a motor accident as a pedestrian. She presented with minimal to moderate traumatic brain injury, with an epicranial hematoma seen on the CT scan. On physical examination, swelling, ecchymosis, loss of function, pain, and bone crepitus of the right leg were observed. AP and lateral X-ray examinations of the right leg showed a comminuted wedge-type fracture of the proximal tibia (Figures 1-2) and a bifocal fracture of the fibula with minimal displacement. The patient reported extreme pain, placing it at a ten on a 1 to 10 pain scale. The patient was placed in a posterior long leg splint and admitted to the Orthopedics and Traumatology ward.

A patient history of hypothyroidism, labile hypertension, type two diabetes and hearing loss was noted. The patient was taking levothyroxine, perindopril/indapamide and rosuvastatin. She was scheduled for surgery one day after being admitted and stayed in the hospital for three days.

**Figure 3:**
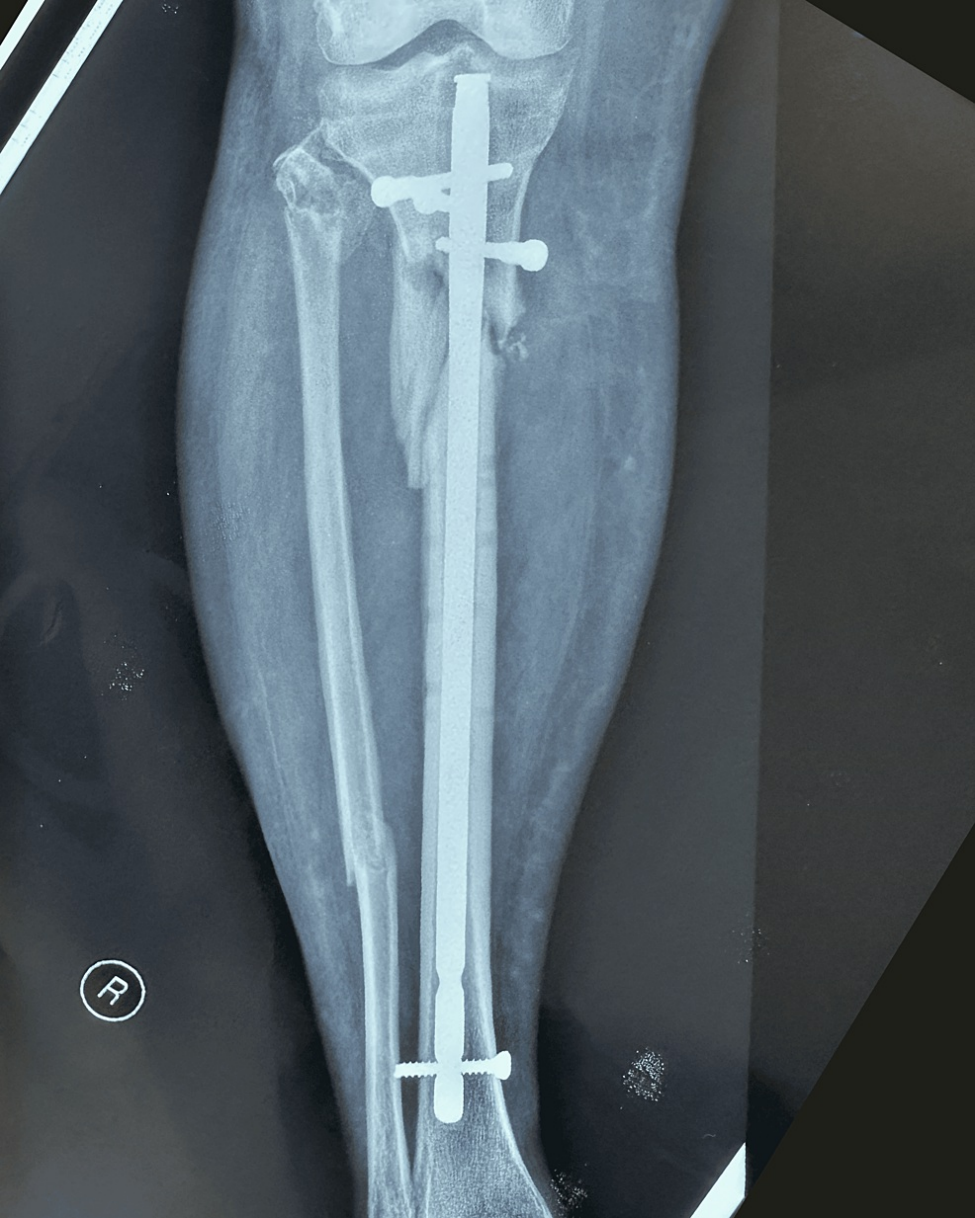
One-month postoperative anteroposterior X-ray of the right leg showing the antegrade tibial nail

**Figure 4:**
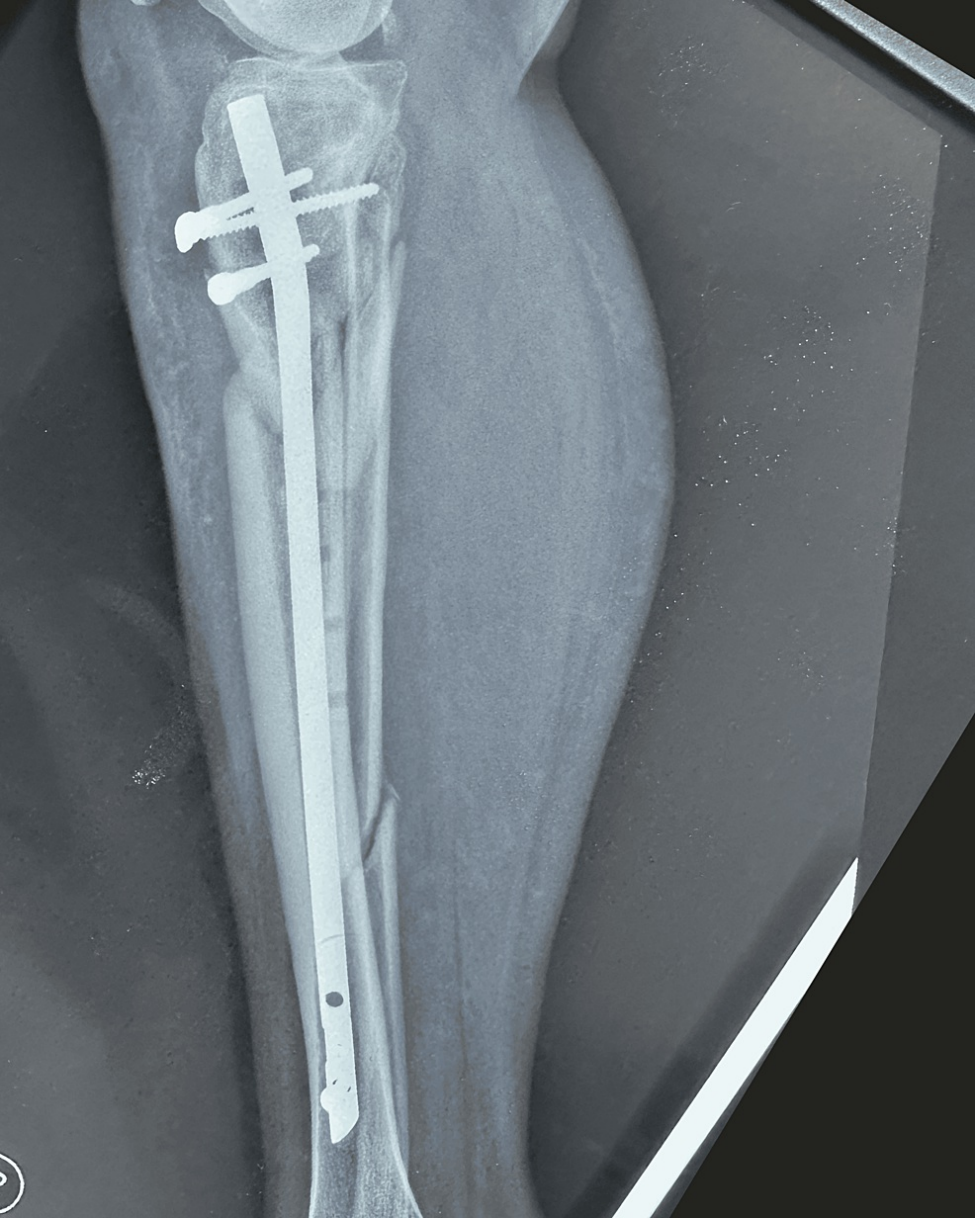
One-month postoperative lateral X-ray of the right leg showing the antegrade tibial nail

The patient returned for frequent follow-ups. At the three-month follow-up, a delayed union was observed and the patient was scheduled for the second surgical intervention: plate removal and conversion to an antegrade intramedullary tibial nail (Figures 3-4).

**Figure 5:**
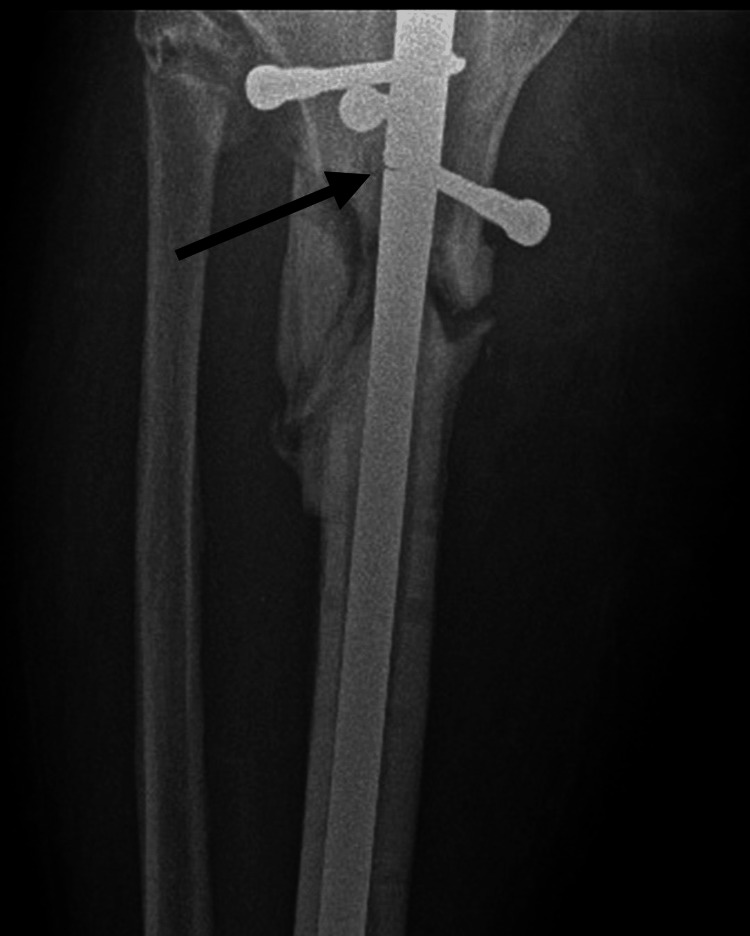
Anteroposterior X-ray seven months after the second surgical intervention, showing oligotrophic nonunion and implant failure (arrow points to the implant failure)

**Figure 6:**
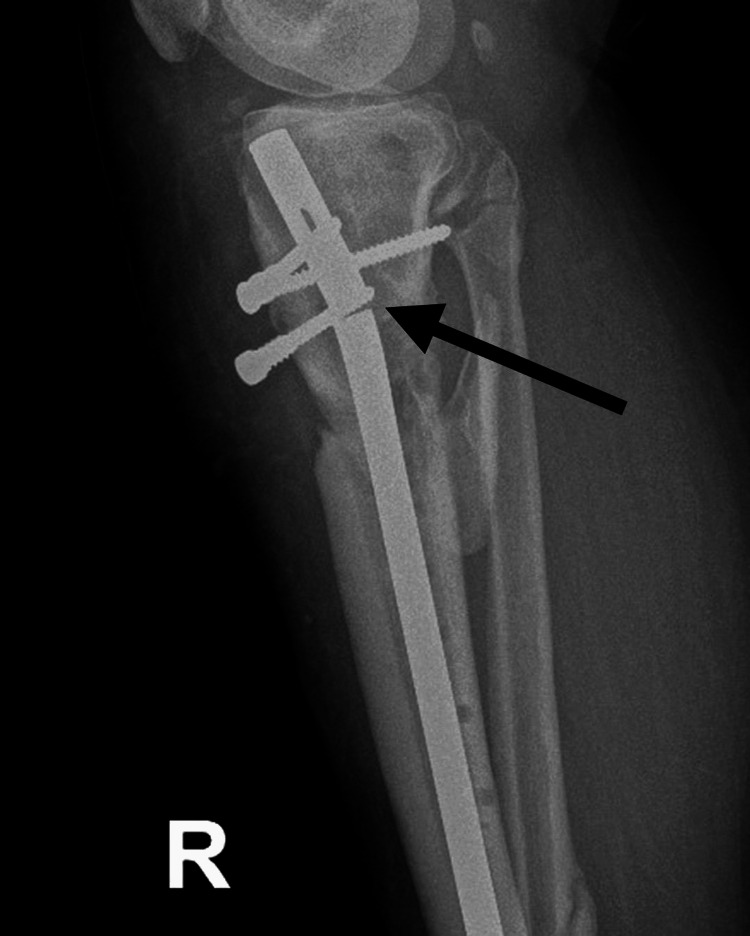
Lateral X-ray seven months after the second surgical intervention, showing oligotrophic nonunion and implant failure (arrow points to the implant failure)

After a period of seven months of progressive weight bearing with external support and physiotherapy, the patient suffered implant failure and was given a diagnosis of oligotrophic nonunion (Figures 5-6).

**Figure 7:**
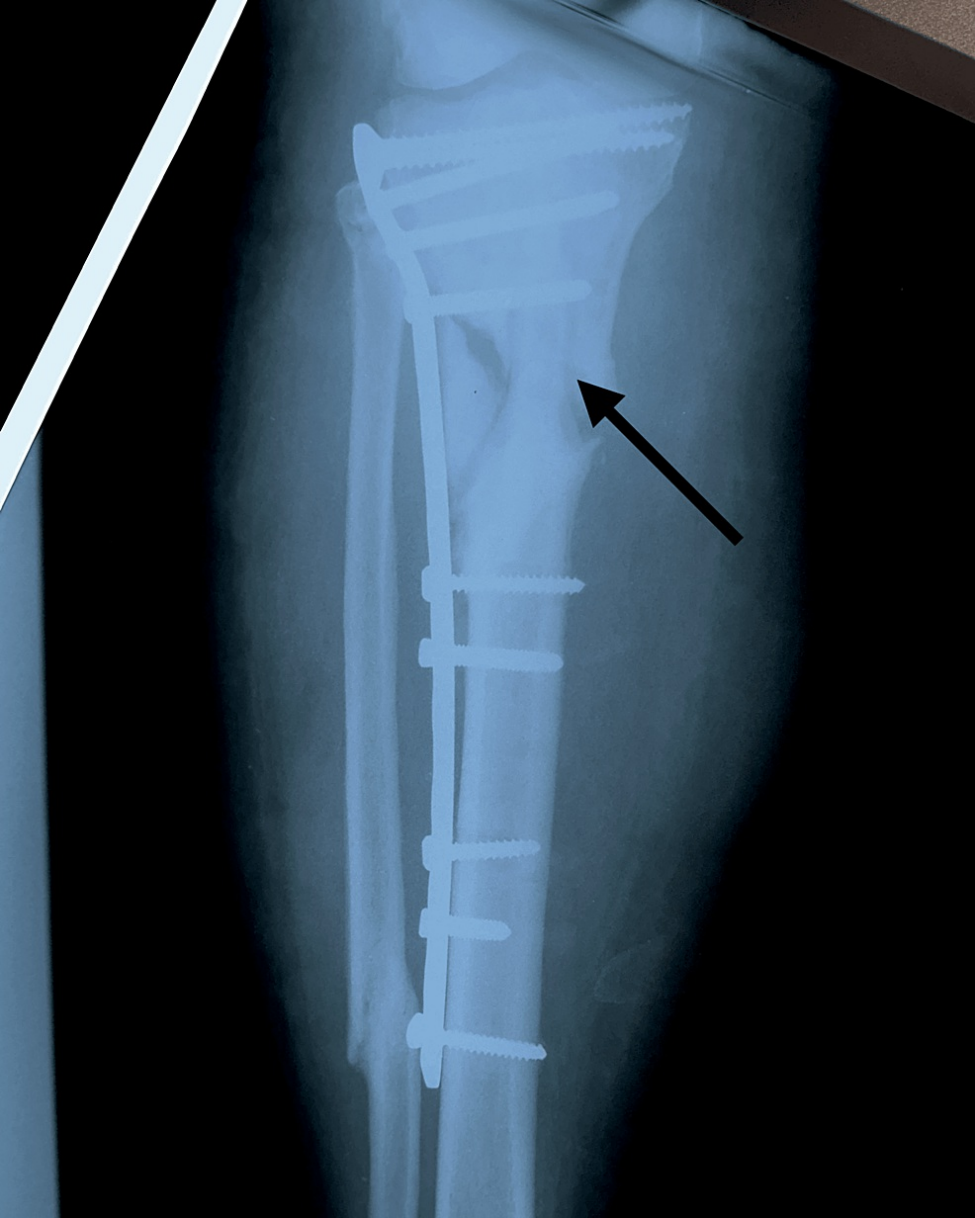
Postoperative anteroposterior X-ray of right leg showing fibular strut graft, proximal tibia locking plate and screws (arrow points to the fibular strut autograft in the tibia medullary canal)

**Figure 8:**
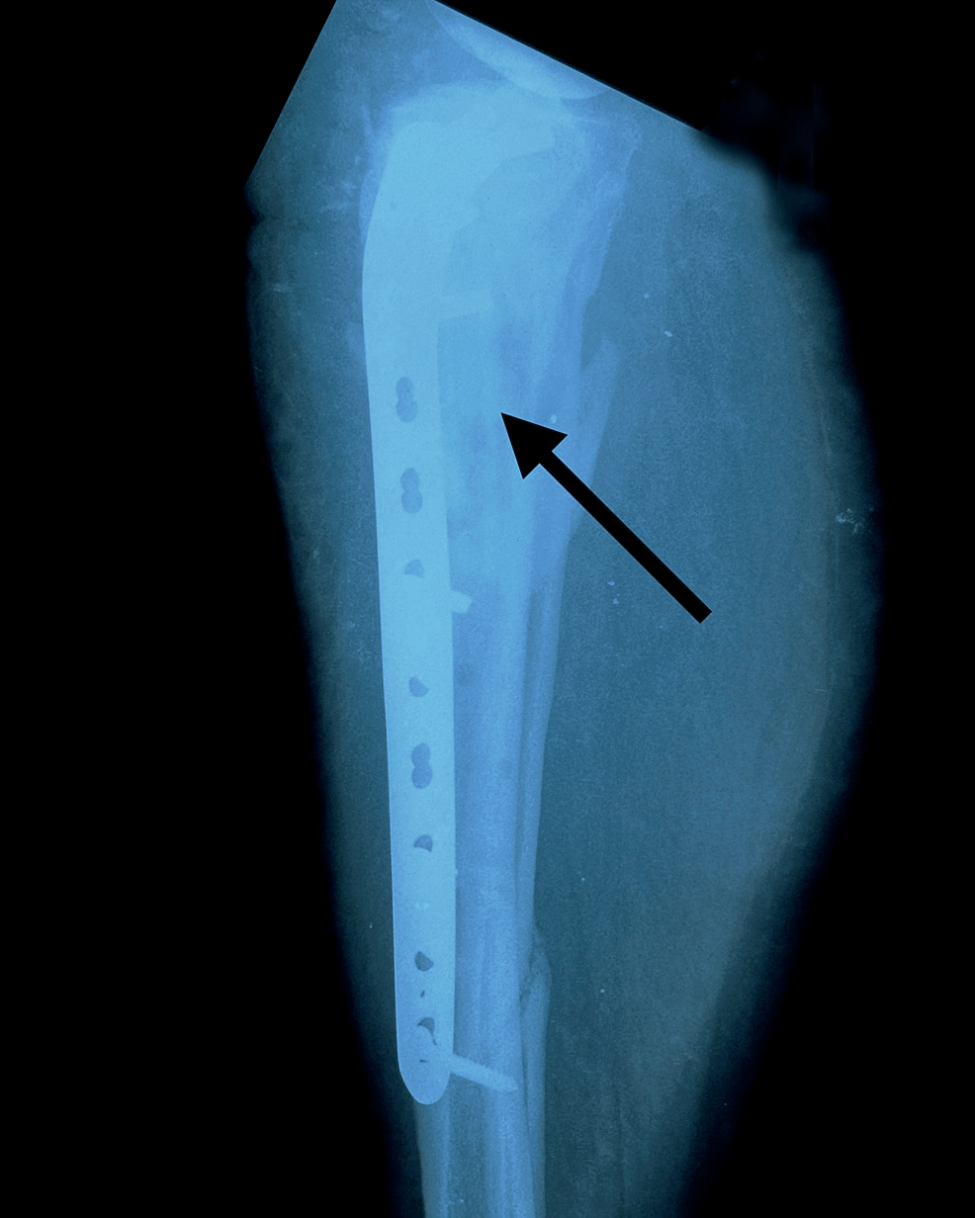
Postoperative lateral X-ray of the right leg showing fibular strut graft, proximal tibia locking plate and screws (arrow points to the fibula shaft strut autograft in the tibia medullary canal)

The patient was scheduled for surgical reintervention one month after the implant failure was diagnosed. At the third and final intervention, the tibial nail was removed and a fibular shaft strut autograft, reamed iliac autograft, and proximal tibia locking plate and screws were used (Figures 7-8).

Two years after the final surgical intervention, the patient had a radiological bone union, could walk without external support for short distances (but preferred a walking stick for longer distances), and reported moderate discomfort at rest and pain when walking for more than 30 minutes. Overall the patient was pleased with the outcome, as she could walk and carry out her daily activities.

**Figure 9:**
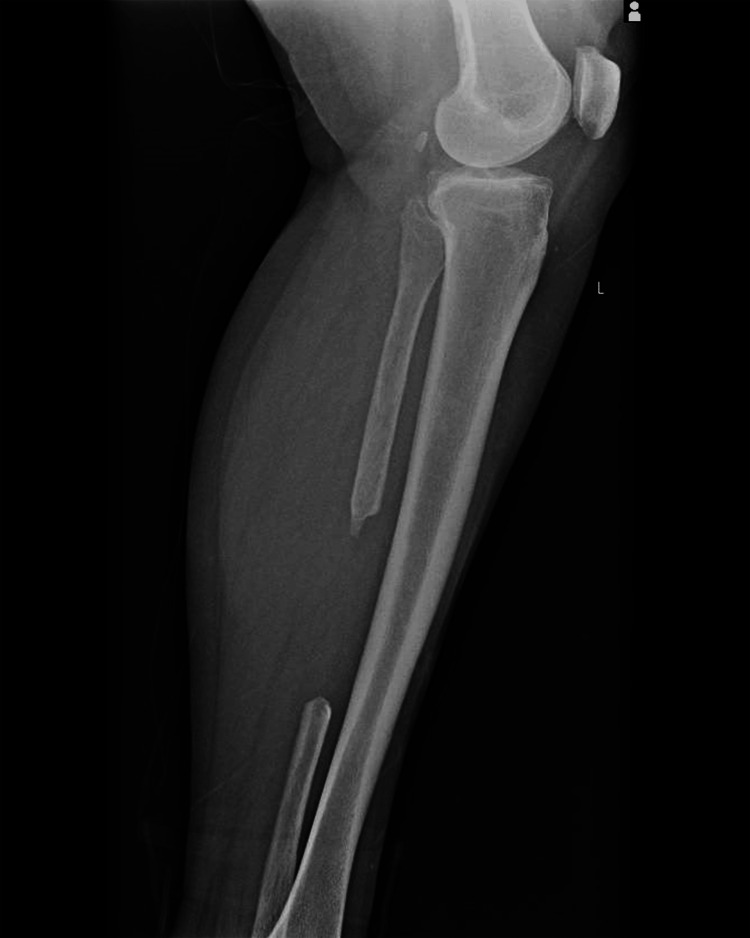
Lateral X-ray of the left leg taken two years postoperatively, with visible fibula shaft graft harvest site shown

**Figure 10:**
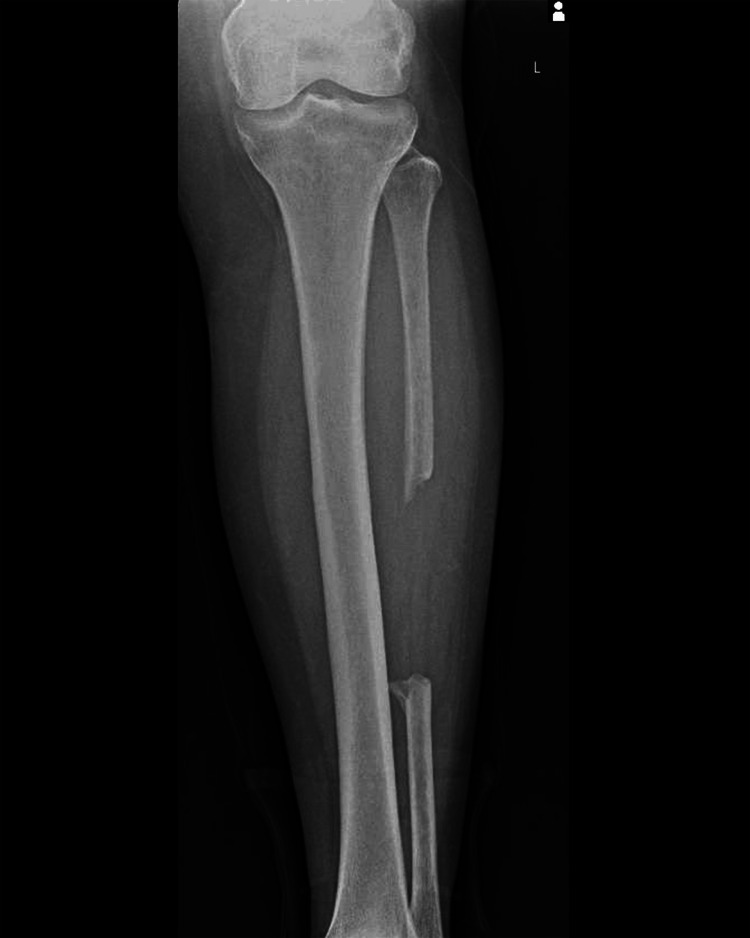
Anteroposterior X-ray of the left leg taken two years postoperatively, with visible fibula shaft graft harvest site shown

For the third and final procedure, the patient received rachianesthesia and was placed on the operating table in a supine position. Antiseptic procedures and appropriate sterile draping were used so as to allow the right leg to be accessed for the purpose of implant removal and reosteosynthesis, the left leg for fibula shaft autograft harvesting (Figures 9-10), and the right hip for iliac crest autograft harvesting. A direct lateral approach to harvesting the fibular shaft autograft from the left leg was used, taking care to avoid the proximal and distal 5 to 10 cm of the fibula. Approximately 8 cm of fibula shaft was harvested. A direct lateral approach was also used to harvest the iliac crest autograft. After extraction, the fibular shaft autograft was stripped of periosteum on the instrumentation table and cut to a suitable length (approximately 7 cm in this case) so that it would fit well in the reamed tibia canal and aid in stabilization and reduction. An acetabular reamer was used to obtain the iliac bone graft.

The deteriorated antegrade tibial nail was extracted and the tibial canal was reamed so as to ensure the proper placement of the fibular graft and as a curative step for the nonunion. The fibular shaft strut graft was inserted through the entry point and along the tunnel where the tibial nail was extracted from; the reamed iliac graft was then spread between the tibial shaft fragments, through an anterolateral approach to the tibia. After the graft placement, the whole construct was fixed using a proximal tibia locking plate and screws, taking an anterolateral approach.

The patient received antibiotic therapy of 1.5 g cefuroxime at the induction of anesthesia, with two more doses of 750 mg given eight and 16 hours postoperatively.

The patient was given the following postoperative rehabilitation recommendations: to receive postoperative physiotherapy immediately (consisting of non-weight-bearing passive and active leg movements for six weeks); then to introduce progressive weight bearing on the limb (5 kg week-on-week increase with external support). The patient reported being able to support 80-90% of her body weight at eight months postoperatively. At 12 and 18 months postoperative, she could achieve full, confident weight-bearing on the right lower limb.

**Figure 11:**
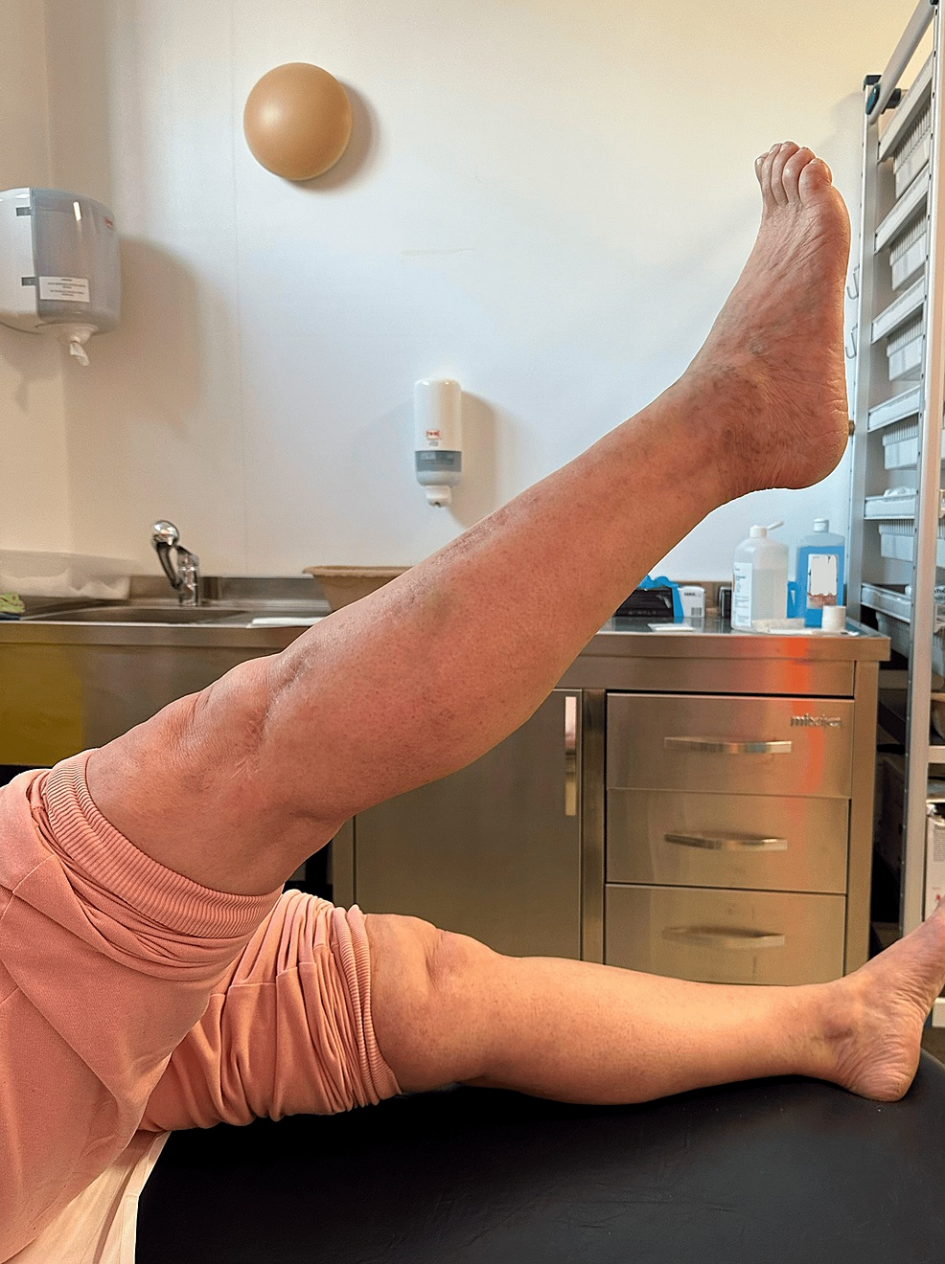
Complete right knee extension at a two-year postoperative follow-up

**Figure 12:**
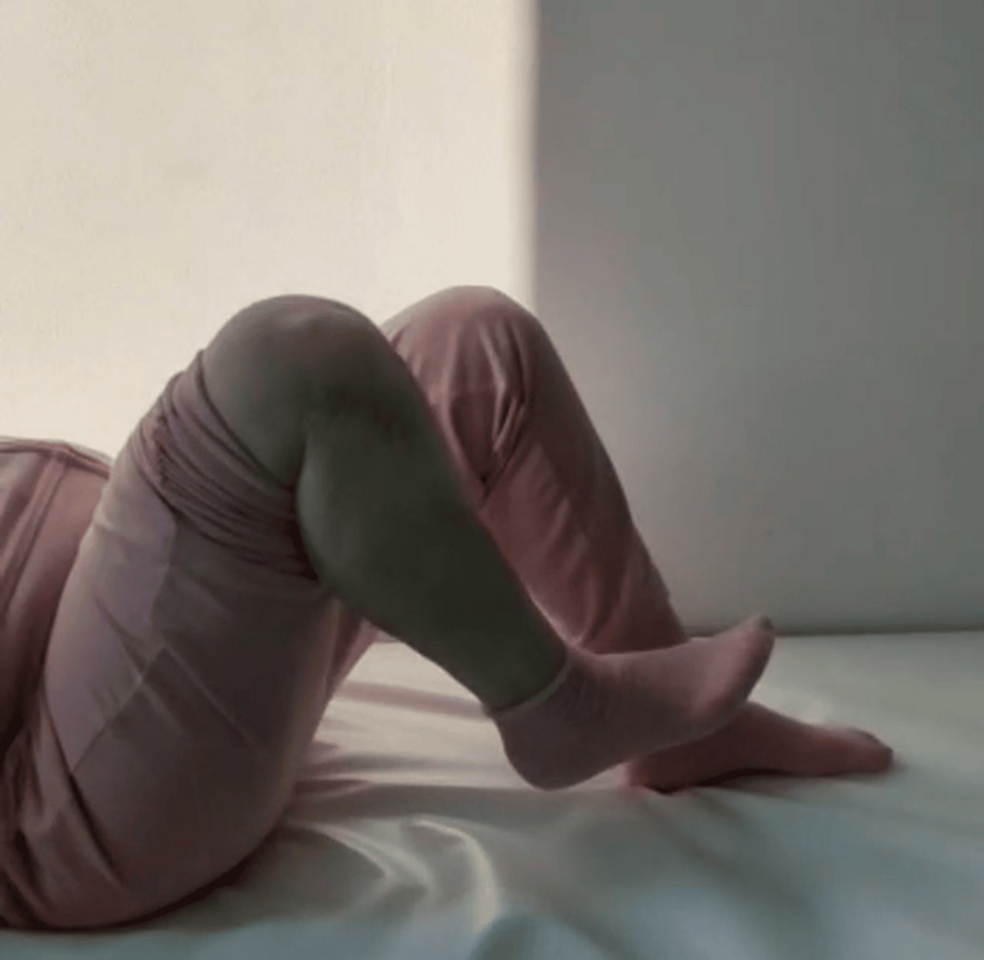
Complete right knee flexion at a two-year post-operative follow-up

**Figure 13:**
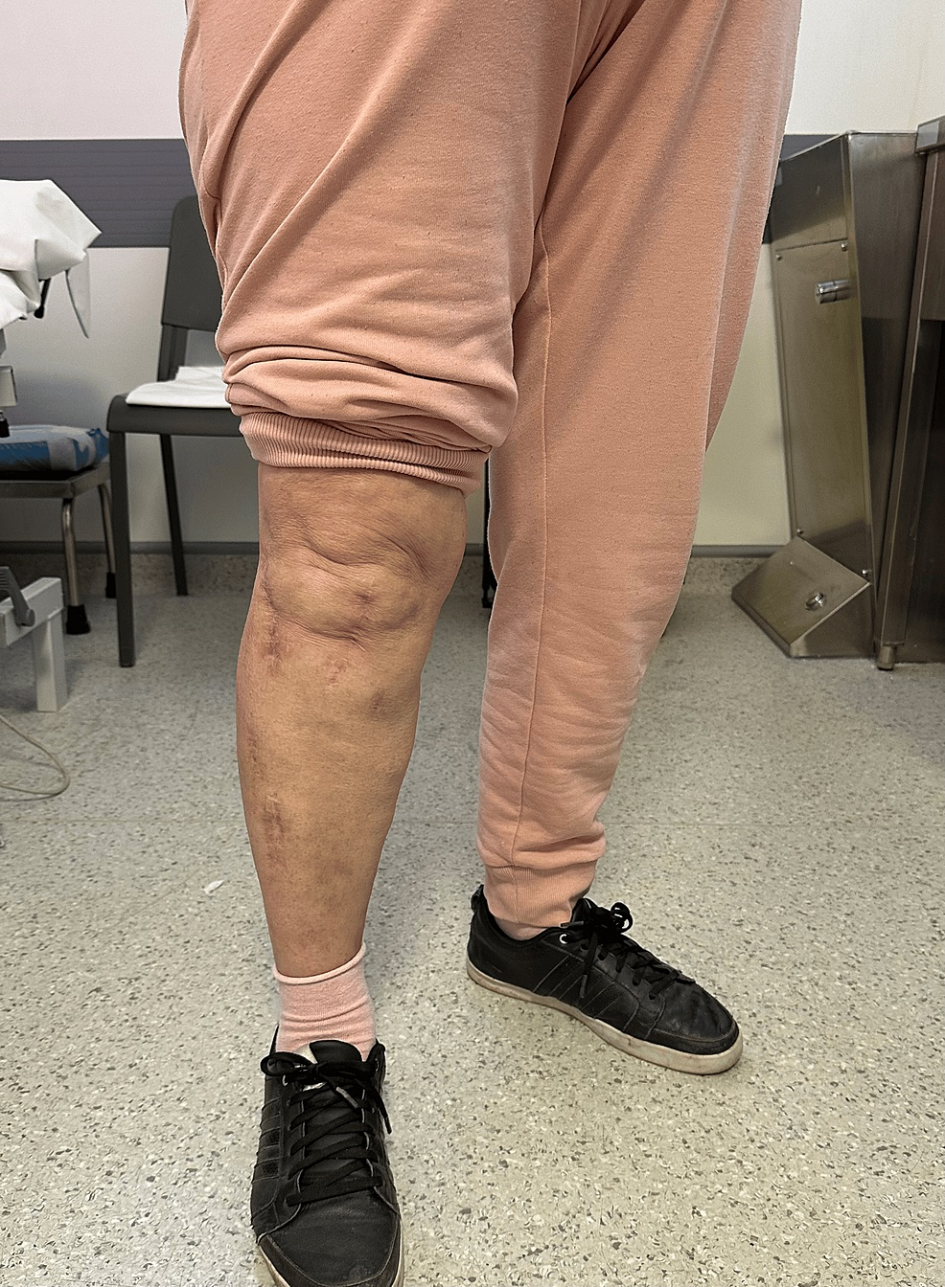
The patient maintaining orthostatism at the time of a two-year postoperative follow-up

**Figure 14:**
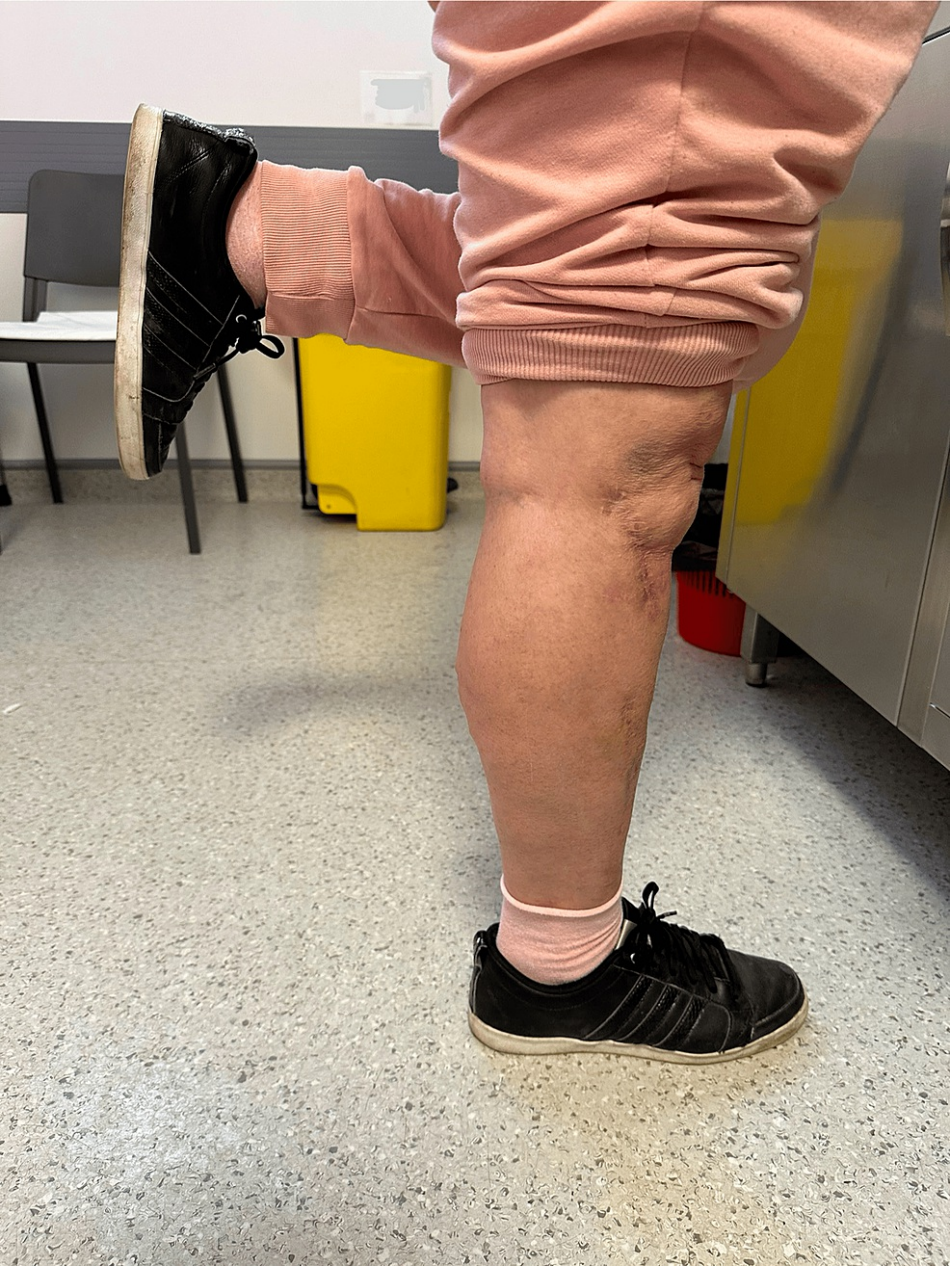
The patient capable of maintaining full-body weight-bearing on the operated leg at the time of a two-year postoperative follow-up

At a two-year postoperative follow-up, the patient presented with painless full ROM (range of motion) in her lower right limb (Figures 11-12), could stand and walk (Figure 13), and could maintain her full body weight on the operated lower limb (Figure 14). The patient reported a dull pain in the knee and proximal right leg, present throughout the day and at rest, and aggravated by walking long distances and maintaining orthostatism for more than 30 minutes. A leg discrepancy of under 2 cm was noted; this was adequately managed with shoe inserts. The patient declared the discomfort and length discrepancy tolerable.

**Figure 15:**
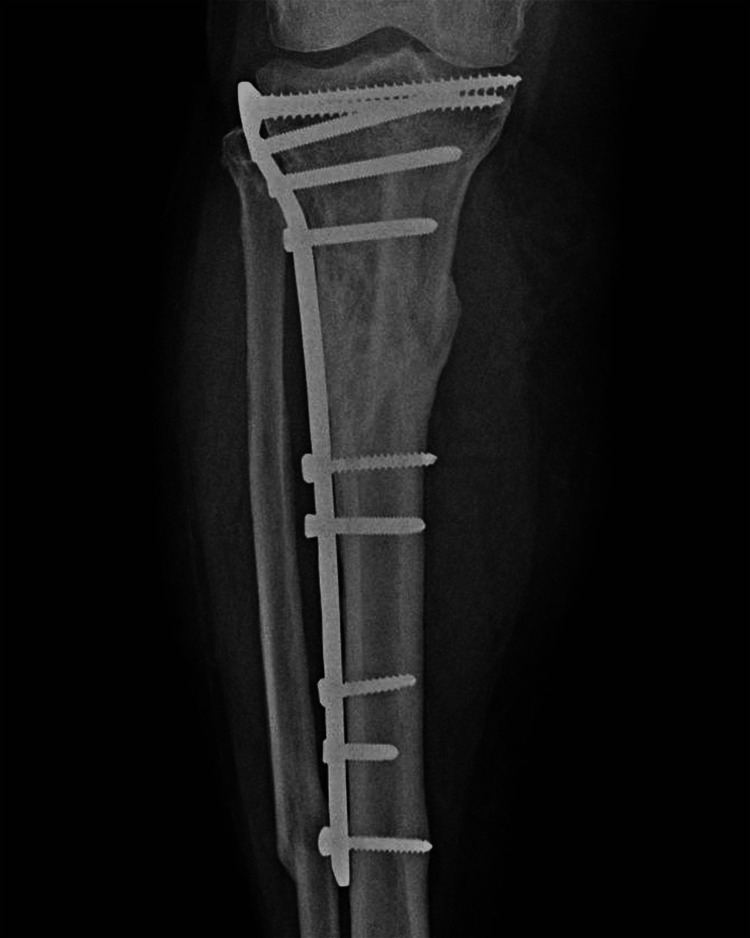
Anteroposterior X-ray of the right leg taken at a two-year postoperative follow-up, showing the bone union and the fibula shaft graft visible in the tibia canal

**Figure 16:**
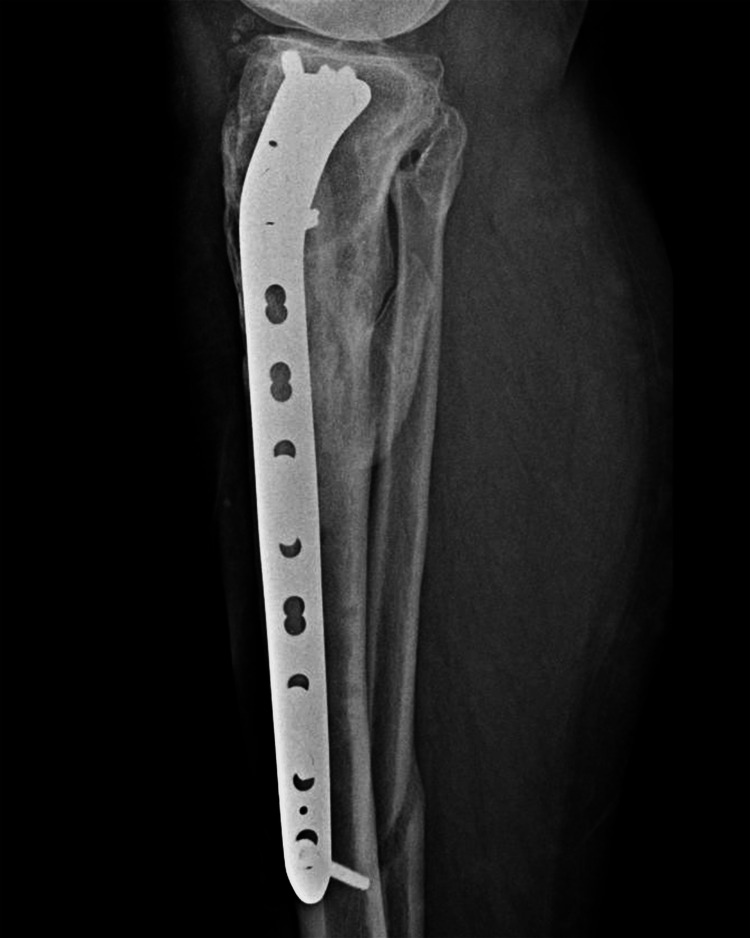
Lateral X-ray of the right leg taken at a two-year postoperative follow-up, showing the bone union and visible fibula shaft autograft in the proximal tibia medullary canal

No pain was reported at the graft harvest sites. Right-leg AP and lateral X-rays taken at the two-year postoperative follow-up show bone union (Figures 15-16).

## Discussion

The most common bone affected by fracture nonunion is the tibia, particularly its proximal third (due to it being poorly covered by tissue and the nature of the forces that result in fractures to this area) [2]. This risk increases as a result of patient comorbidities, with patients with a history of diabetes being seven times more likely to develop fracture healing problems [5]. In a survey of orthopedic surgeons, 59% of them agreed that diabetes played a major role in fracture nonunion [6].

A nonunion can be defined either by its inability to form a bridging callus without additional intervention [5] or by the time it takes to show progress in union: either no union at up to eight months for simple fractures or up to nine for complex ones [4]. Another definition for nonunion is no healing seen on imagistic investigations at three months or no union at nine months [5].

Open fractures are shown to lead to nonunion more often than closed fractures due to the higher risk of infection or the degree of damage to the blood supply and periosteal stripping [6]. Soft tissue damage is also shown to happen in closed fractures such as cases of high-energy trauma [6]; the patient in this particular case suffered from high-energy trauma caused by a traffic accident.

The treatment of fracture nonunion is guided by five standards: management of adjustable risk factors, solid fixation, mechanical alignment, mechano-biological triggering, and prompt rehabilitation [5]. In the case of oligotrophic nonunion, it is best to consider enhancing both the mechanical and biological aspects of bone healing [6].

In light of these factors, the patient's diabetes was more rigorously managed; she went from a blood sugar level of 140 mg/dl at the time of the first surgical intervention to a level of 112 mg/dl at the time of the last surgical intervention. At the third surgical intervention, fibular and reamed iliac crest grafts were used as structural and biological adjuvants, and a prompt rehabilitation regime was instituted. Postoperative physiotherapy was introduced immediately, with progressive weight-bearing starting six weeks postoperatively (consisting of a 5 kg week-on-week weight-bearing increase on the operated leg). The patient managed to bear between 80% and 90% of her body weight at eight months postoperative.

In addition to her diabetes, the patient monitored her other endocrine comorbidities (namely her hypothyroidism and levothyroxine dosage) more closely, making regular visits to the endocrine specialist.

The general treatment options for nonunion are either non-operative or operative [6]. Non-operative treatment is employed when the biological status of the bone and union progress cannot be assessed and it may only be a matter of time until fracture union [6]. If radiological examinations over time show no evolution, then surgical treatment is required [6]. Nonsurgical treatment is classified either as indirect or direct intervention [6]. Indirect non-operative solutions are to address smoking and nutrition, promote the better management of metabolic and endocrine comorbidities (in this patient's case, her diabetes and hypothyroidism), and ensure the adequate management of certain medications (in this patient's case, her levothyroxine dosage was more closely monitored by an endocrine specialist) [6]. The direct non-operative management of nonunion involves mobilization in conjunction with interventions such as introducing weight-bearing, external support, ultrasound or electromagnetic stimulation, and hormone adjuvants such as teriparatide (a synthetic form of parathyroid hormone) [6].

Surgical treatment is chosen when no evolution in bone healing is seen following the use of non-operative methods. Surgical options for treating the nonunion of tibial shaft fractures include nailing, nail dynamization, reaming and exchange-nailing with a larger diameter nail, plating, or the use of an external fixator [6].

Care must be taken when considering the timing of any reintervention. Giving a diagnosis of nonunion too early might make the patient suffer unnecessary surgical interventions, while a diagnosis that comes too late might prolong the patient's physical, psychological, and socioeconomic distress [6]. A period of six months is often enough to evaluate the status of tibia shaft fractures [6]. Our patient was monitored for eight months after her tibia was reamed and nailed (an intervention aimed at treating the delay in union of her fracture). During this time, she underwent physiotherapy and attempted progressive weight-bearing until her implant failure was diagnosed. Intramedullary nailing was chosen for the treatment of delayed union after primary plating, as it provides stable fixation with minimum additional soft tissue insult [5]. Reaming can also help in depositing bone graft and stimulating healing; its success is dependent on the extent of the bone defect [6].

A nonunion repair using plating can provide a stable and aligned construct with adequate compression [5]. Plating, coupled with added intramedullary fixation with a fibula shaft strut autograft and reamed iliac autograft added to areas of bone defect and comminution, resulted in a favorable outcome in our particular case.

Bone autografts are considered the gold standard in fracture nonunion [5,6]. Common cancellous bone graft harvest site choices include the iliac crest, distal femur, olecranon, proximal humerus, and proximal and distal tibia [6]. It was determined that the iliac crest autograft used in femoral and tibial non-unions has a success rate of over 90% [6]. In this case, the fibular strut autograft was used to bridge the comminution and provide intramedullary stabilization. The fibula strut graft can also be used in the proximal humerus for fractures with marked comminution and medial instability [7]. The literature showed that non-vascularized fibular shaft autografts are less likely to successfully treat nonunion than vascularized fibular grafts [8]. However, a non-vascularized fibular shaft strut autograft was successful in this case.

## Conclusions

A proximal tibia locking plate, used together with non-vascularized fibular shaft strut autograft and reamed iliac crest autograft was used to successfully treat an aseptic oligotrophic nonunion in a comminuted high-energy tibial metaphyseal fracture with implant failure in the case of a 62-year-old patient with multiple comorbidities.

At a two-year postoperative follow-up, the patient had radiological signs of complete union, full range of motion, and could walk and support her full body weight on the operated leg.
